# Supplementary material for: Assessing the Core Variables of Business Managers’ Intuitive Decision Ability: A Review and Analysis
Source: Behav Sci (Basel). 2022 Oct 24;12(11):409. doi: 10.3390/bs12110409 (PMC9687419; doi:10.3390/bs12110409)
Supplement: Supplementary file 1 [file behavsci-12-00409-s001.zip › behavsci-1903858-supplementary.pdf]

SUPPLEMENTARY INFORMATION ……

Details of the survey questions used for calculating the level of a decision maker's business related phenotype (BRP), training and experience being variables for use in the research paper

'Assessing the core variables of business managers' intuitive decision ability… review and analysis'.

With the variation in business types it was necessary to use an approach that was general. Discussions with experts suggested seeking views on the issues which face managers was the most practical and efficacious approach. Accordingly, other than obvious and simple cardinal defining variables, the survey questions are based on obtaining each manager's view on the truth of a number of business issue statements. Respondents were asked to rate each statement on a truth 1 to 5 scale (Likert). For questions not in this general category the value requested is defined in the questions as presented.

Listed below are all the variables sought for each core postulate. The value in brackets after each question/statement is the one factor solution value (component matrix) which lead to the attribute (phenotype, training and experience) value for each manager.

In addition, the truth of statements covering a managers/business' objectives were sought. The three factor component matrix values for each question are similarly provided at the end of each statement in brackets.

Rather than use standard personality, and Locus of Control, tests, given the objective was to assess personal factors defining a business manager's approach (BRP), the questions were formulated around business issues with the same basic ideas as used in conventional tests. These reflect what might be called 'management style' expressing a business manager's personality and control attitudes with respect to business decision making.

All the questions have been tested and used many times in managerial work and research (see, for example, FBM… The Human Factor, 2nd ed, CABI, Wallingford, UK., 2019: and Psychometric testing for assessing farmers' managerial ability, Paper to the NZ Agricultural and Resource Economics Society, 2006 DOI: 10.22004/ag.econ.31951)

Survey questions for….

<u>Business Related Phenotype…</u>

Intelligence and related

For the LAST year of formal study, what was your average grade (as you recall) %? (.050)

If managers were rated on a 10 (excellent) to 1 (poor) scale for managerial ability, at what level of skill rating would you give yourself? (.013)

Business Related Personality as reflected in 'management style'.

You find it easy to ring up strangers to find out technical information (-.109)

Where there are too many jobs for the time available you sometimes become anxious (.710)

You tend to tolerate mistakes and accidents that occur with employees and/or contractors (-.007)

You share your successes and failures with other managers in similar business (.129)

You admire managers who are financially logical and don't let emotions colour their decision (.183)

You sometimes don't sleep at night worrying about decisions made (.671)

You tend to worry about what others think of your methods (.518)

You are happy to make do with the materials you have to hand (.112)

You normally don't rest until the job is fully completed (.329)

When the pressure is on you sometimes become cross and short with others (.590)

You are inclined to let employees/contractors do it their way (-.064)

You not only speak your mind and ask questions at business meetings but also enjoy the involvement (-.018)

It is important to earn the respect of business colleagues in the local community (.297)

Maintaining a presence in local community activities is important (.049)

Business control attitudes as related to the Locus of Control

While being a good manager involves some training, experience and reading, management skill is mainly determined by your genes (.222)

You can work hard at creating good relationships between nearby managers, but often your efforts fall on deaf ears as people are commonly uncooperative and self interested (.262)

In local body affairs it's easy for a hard working and dedicated individual to have an impact in getting changes for the better (.051)

Often I get frustrated as circumstances beyond my control impede the smooth progress of my management plans and decisions (.532)

I seldom change my management and production systems unless I'm doubly sure the change will be positive. So much depends on chance (.374)

When I know I'm right I can be very determined and can make things happen (.209)

General

Age (1= <=25 years, 2=26-35, 3=36-45, 4=46-55, 5=56-65, 6=>65 years) (.116)

Training …

Covering educational achievement and the approach/action regarding learning about issues likely to be faced …

What was the level at which you stopped formal education? (-.105)

Primary school 1; secondary school up to 3 years 2; secondary school – 4 or more years 3; tertiary education – up to 2 years 4; tertiary education – 3 or more years 5.

For most things you seek the views of many people before making changes to your operations (.373)

Keeping records on just about everything is very important (.605)

You tend to write down options and calculate monetary consequences before deciding (.506)

It is very important to stick to management principles no matter what the pressure to do otherwise (.472)

Attending business and manager meetings is vital (.526)

Proper retirement is a major consideration (.495)

It is very important to improve the condition of the physical assets (.459)

So far I have largely managed to achieve my goals (.327)

I reckon 'good luck' doesn't exist – 'luck' is really good management and 'bad luck' poor management (.295)

I tend to carefully plan ahead to ensure my goals are achieved, and often do budgets and commit my ideas to paper (-.078)

Experience …

Covering approaches to experience and the lessons learnt…

Years of experience (in the business) (-.237)

You tend to mull over decisions before acting (.105)

You usually find discussing everything with members of your family and/or colleagues very helpful (-.262)

You find investigating new production methods exhilarating and challenging (-.599)

You find talking to others about production ideas stimulates and excites you as well as increasing enthusiasm for new ideas (-.571)

Having to make changes to well established management systems and rules is a real pain (.462)

You normally enjoy being involved in business organisations (-.360)

You sometimes believe you are too much of a stickler for checking and double checking that everything has been carried out satisfactorily (.062)

You generally choose conclusions from experience rather than from hunches when they are in conflict (-.147)

You are much happier if everything is planned well ahead of time (-.046)

Developing facilities and systems that give good working conditions is crucial (-.382)

It is very important to ensure employees enjoy their jobs (-.430)

While I don't particularly enjoy my business, I carry on as I don't have a background that allows shifting into another occupation (.446)

I never try anything that might not work (.375)

I'm using exactly the same production methods that I have used for many years because they have stood the test of time (.586)

It is no use being stubborn about a job or management approach that doesn't initially work (-.012)

It is safer not to rely on others to get the job done well and on time (.478)

I'm able to get others to do the jobs my way (-.263)

Too often I end up having to run the business to suit others' demands (.307)

I find most employees work hard and finish tasks set very adequately after a bit of training where necessary (-.431)

The years where the business has shown poor production and/or profit have been due to circumstances totally out of my control (.183)

Some people seem to be just lucky and everything works out for them, but it hasn't happened to me much (.491)

When things go wrong it is so often due to events beyond my control – the weather intervenes, the market chosen has a sudden price dip….. (.357)

Notes

The question sets were developed after reviewing the five factor personality theory (Matthews, G., & Deary, I. 1998. Personality traits. Cambridge University Press), the Locus of Control theory (Rotter, J. 1966. Generalized expectancies for internal versus external control of reinforcement. Psychological Monograph, 80(1):1-28), the high correlation between formal grades and intelligence quotient (Deary, I., Strand, S., Smith, P., & Fernandes, C. 2007. Intelligence and educational achievement. Intelligence 35(1): 13-21), business consultants and practitioners. They were also pretested with a number of managers with appropriate corrections made. The questionnaires were vetted and approved by a university ethics committee after completing requested modifications. As noted, the questions have all been tested and used several times in research work… as an example see DOI:10.22004/ag.econ.31951

Questions selected from the surveys to obtain each manager's business objectives

The managers were asked to rate the truth of each of the following statements using a 1 to 5 scale (Likert Scale). The numbers in brackets after each statement are the factor contributions for a three factor solution (Varimax rotation). These provide the weightings for calculating the modified cardinal objectives used in judging each respondents achievement level. The categories of objective types are based on various assessments of alternatives (for example, Gasson, R., Crow, G., Errington, A., Hutson, J., Marsden, T., & Winter, D. 1988. The farm as a family business, a review. Journal of Agricultural Economics, 39:1-41.)

It is very important to pass the business to family members (.654, -.249, .276)

It is very important to reduce risk using techniques like diversification, conservative production systems, keeping cash reserves…… (.066, .174, .662)

Making a comfortable living is important (.160, .413, .304)

It is necessary to keep debt as low as possible (-.040, -.066, .775)

It is essential to plan for reasonable holidays and plenty of leisure time (-.021, .654,.066)

Doing the jobs that I enjoy is a very important part of the operation (.093, .335, .382)

Minimising pollution is very important (-.006, .441, .348)

I enjoy experimenting with new products and production systems (.166, .594, -.048)

You must always be striving to increase the total value of assets (.652, .351, -.123)

Constantly expanding the size of the business is absolutely necessary (.697, .154, -.318)

Aiming for maximum sustainable net cash returns is very important (.474, .441, .058)

Giving assets to the children so they can pay for education and/or set up businesses is very important (.563, .022, .241)

The factor component matrix values make it clear which variable combinations make up the important basic objectives (i.e. the main objective categories involve profit, leisure, risk, sustainability, inheritance, work enjoyment, growth and pollution).

Additional information

Monographs giving full details of the base surveys (background, sampling, question development, tables of all the data and descriptive analyses) are available on line at ….

URL's will be provided after review for anonymity purposes as required by Journals.
